# Total Mercury Mass Load from the Paglia–Tiber River System: The Contribution to Mediterranean Sea Hg Budget

**DOI:** 10.3390/toxics10070395

**Published:** 2022-07-16

**Authors:** Silvia Fornasaro, Guia Morelli, Pilario Costagliola, Valentina Rimondi, Pierfranco Lattanzi, Cesare Fagotti

**Affiliations:** 1 Department of Earth Sciences, University of Pisa, Via Santa Maria 53, 56126 Pisa, Italy; silvia.fornasaro@unipi.it; 2 Consiglio Nazionale delle Ricerche, Istituto di Geoscienze e Georisorse-IGG, Via G. La Pira 4, 50121 Florence, Italy; pierfrancolattanzi@gmail.com; 3 Department of Earth Sciences, University of Florence, Via G. La Pira 4, 50121 Florence, Italy; pilario.costagliola@unifi.it (P.C.); valentina.rimondi@unifi.it (V.R.); 4 ARPA Toscana, Area Vasta Sud, Loc. Ruffolo, 53100 Siena, Italy; c.fagotti@arpat.toscana.it

**Keywords:** mercury, mass load, particulate matter, bedload trap, Monte Amiata mining district, Mediterranean Sea

## Abstract

The Mediterranean Sea is characterized by a marked mercury (Hg) geochemical anomaly, arising in part from large Hg deposits. Mercury mass loads discharged from the Monte Amiata mining district (Central Italy) to the Mediterranean Sea through the Paglia–Tiber River system were estimated. Data from two seasons showed that up to 40 kg year^−1^ of Hg are drained to Tiber River and finally to the Mediterranean Sea. The mercury mass loads varied in different seasons, from 3 mg day^−1^ in the upper section of Paglia River in November to 42 g day^−1^ before the confluence with Tiber River in June. Along Tiber River, up to 15 ng L^−1^ of the total Hg found at a site after Rome showed that Hg can be discharged to the sea. The Alviano reservoir along Tiber River acts as a temporary trap for Hg-rich particulate, while dam operations may promote Hg release (up to 223 g day^−1^). The combination of hydrologic factors controlling Hg transport, the torrential regime in the upper catchment of Paglia River, the waterway steepness, together with Hg-contaminated legacy sediments in the Paglia River floodplain, make the Paglia–Tiber River system a long-lasting intermittent source of Hg to Tiber River and the Mediterranean Sea.

## 1. Introduction

The global present-day mercury (Hg) discharge from rivers to oceans is estimated to be 27 ± 13 Mmol year^−1^ (5500 ± 2700 Mg year^−1^), of which 28% reaches the open ocean, while the rest is deposited to ocean margin sediments [1]. Besides atmospheric inputs and direct discharges of wastewater effluents, rivers represent important contributors of Hg to the sea, carrying Hg mainly through mine drainage and runoff from contaminated wastes of abandoned mining sites [2,3,4,5,6,7]. The Mediterranean Sea is characterized by a wide Hg geochemical anomaly [8,9]. The contribution of Hg discharge into the Mediterranean Sea from large rivers (i.e., Po, Isonzo, Rhone, Ebro, and Nilo Rivers) was estimated at between 8 and 13 t year^−1^ [8,10], with, for example, Hg discharges from Rhone River reaching up to 572 kg year^−1^ [11]. However, this estimate did not include most of the rivers draining the important Hg mining districts, although the largest cinnabar deposits of the world (Almadén, Spain; Idrija, Slovenia; and Monte Amiata, Italy, concentrating approximately 65% of global Hg resources) [8,12] occur in the Mediterranean region [13,14].

Several studies [15,16,17] highlight that a detailed estimate of the Mediterranean Hg budget should include Hg delivered by streams draining Hg mining districts. For example, about 80 kg year^−1^ was estimated by Faganeli et al. [16] from Isonzo River, draining the Idrija mine district, and a net export of approximately 115 kg year^−1^ was calculated from the Marano–Grado Lagoon (polluted from the Idrija mine) to the Adriatic Sea [18]. Tiber River, the main river of Central Italy, flowing through the city of Rome, receives waters from Paglia River, which drains the Monte Amiata mining district (MAMD; southern Tuscany, Italy), the third-largest Hg-producing district worldwide. Today, after more than forty years from the closure of mining and smelting activities in the area and the partial reclamation of the main mining areas (Siele and Abbadia San Salvatore) [19], Hg is still persistent in the environment as a result of the widespread presence of polluted stream sediments along rivers draining the MAMD [7,20,21,22,23,24,25].

The Paglia–Tiber River system represents, therefore, the main transport route for Hg from the MAMD to the Mediterranean Sea. Indirect or partial estimates about the Tiber River Hg mass loads were reported by Cossa et al. [8], Montuori et al. [9], and Rimondi et al. [20]. Rimondi et al. [20] provided a first estimate of the mass load (mass/time; e.g., g day^−1^ or kg year^−1^) of Hg carried by the waters of Paglia River and Pagliola Creek, draining the Abbadia San Salvatore mine (ASSM), the most important mining site of the MAMD. They suggested that up to 11 kg year^−1^ of Hg can be discharged by Paglia River into Tiber River, a few kilometers south of the border between Tuscany and Umbria regions, by extrapolating to a yearly scale three measurements conducted between March 2011 and March 2012 [20]. However, the results showed that the input of Hg to Tiber River is not constant but intermittent and widely variable because of the torrential regime of tributaries. The variability ranges from about 1 g day^−1^ in the dry season to 32 g day^−1^ in the wet season. These estimates would correspond to 0.3 and 12 kg year^−1^, respectively. In addition, the authors highlighted that the annual Hg load inferred for the Paglia River catchment should be considered a conservative estimate because it was measured during baseline (low) flow conditions. A new spot survey was carried out in September 2014, including three locations along Tiber River, but the results were considered preliminary and were published only in abstract form [26]. Therefore, the annual amount of Hg discharged by Tiber River into the Mediterranean Sea is far from precisely known. The Water Framework Directive [27] commits European Union member states to achieve a good ecological status of water bodies. Riverine sediment and water conservation are a priority due to the importance of this environmental compartment in the transport and storage of trace metals at the catchment scale. Moreover, human health concerns associated with the presence of high levels of inorganic Hg are associated with the production of its most toxic form, methyl-Hg, occurring in reducing zones in freshwater and terrestrial environments. Exposure to humans and wildlife caused by its bioaccumulation include neurotoxicity associated with methylmercury entering the bloodstream, damage to fetuses, disabilities, and reproduction interferences (e.g., Refs. [1,14,15]).

The remediation interventions in the MAMD and constant monitoring of Hg concentrations in the basin are important tools for safeguarding the well-being of the aquatic and marine ecosystem, but they are not a definitive solution. Mercury contamination in the Paglia–Tiber River system currently represents an important environmental issue at the national scale. It brought together the environmental agencies and the local authorities of the regions involved (Tuscany, Lazio, and Umbria) to define an adequate strategy of intervention and environmental remediation to decrease any potential risk for human health. In this context, the Tuscan regional environmental agency (ARPAT), with the scientific support of the University of Firenze, carried out in 2020 a detailed study of Hg loads transported in the Tuscan segment of Paglia River, including two right-side tributaries (Siele and Stridolone Creeks) draining the mining areas of Siele and Cornacchino. The additional purpose of the study was to quantify the contributions from these two creeks.

We report here the results of this study, and we include data collected in Tiber River during the survey in 2014, with the aim of providing an improved assessment of the mass load of Hg transported from the MAMD to the Mediterranean Sea through the Paglia–Tiber River system. As noted above, the preliminary 2014 campaign was intended to provide a snapshot of the entire Paglia–Tiber system. Therefore, many points of the two campaigns (2014 and 2020) do not overlap. However, we believe that the combined data make a consistent contribution to estimate the total amount of Hg delivered into the Mediterranean Sea by waterways draining abandoned mining sites.

## 2. Materials and Methods

### 2.1. Study Area and Sampling

#### 2.1.1. Study Area

**Figure 1:**
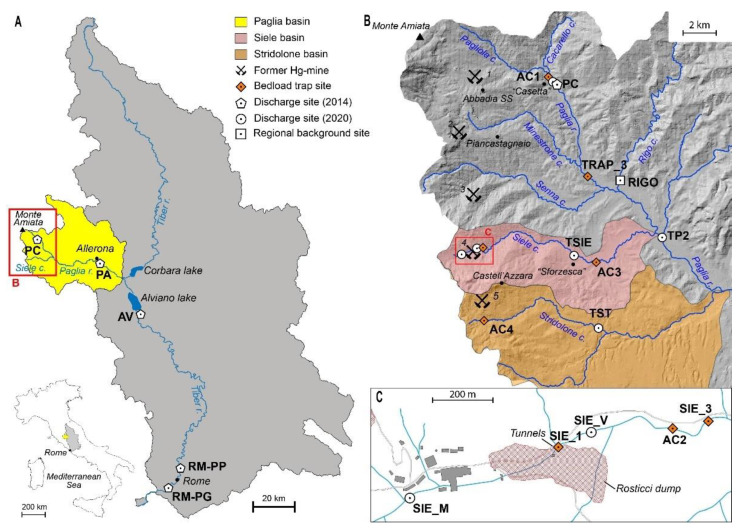
Map of the study area and location of the sampling sites. (**A**) Paglia–Tiber River basin; (**B**) upper part of the Paglia River basin, the Siele (pink area), and Stridolone (orange area) Creek basins; (**C**) details of the Siele mining area. The main mining sites are also reported: (1) Abbadia San Salvatore (ASSM), (2) Case di Paolo, (3) Senna, (4) Siele, (5) Cornacchino.

The Paglia–Tiber River system is located in Central Italy (Figure 1). Tiber River is the third-longest (405 km) river in Italy, with an annual mean flow rate of 230 m^3^ s^−1^. The source is in the Apennine mountains. The catchment area is about 17,400 km^2^ and receives an average of about 95 cm year^−1^ of rain (period 1917–2008; [28]).

Its fluvial basin hosts large areas devoted to agriculture and key industrial activities for Central Italy. In the lower course of Tiber River, after the confluence with the major tributary, Aniene River, the water quality decreases dramatically due to the contributions of industrial districts, several ditches, heavily urbanized watersheds, and agricultural wastewaters [29,30,31].

The Tiber River Valley runs along an extensional basin, developed since the late Early Pliocene, whose direct faults crosscut thick sequences of sedimentary and volcanic successions [32]. Along Tiber River, there are several artificial reservoirs. The widest is the Alviano reservoir (12,000,000 m^3^; [33]), located soon after the Paglia–Tiber confluence and built in 1963 for electricity production.

Paglia River is one of the most important right-side tributaries of Tiber River, discharging into Tiber River south of Orvieto (Umbria), about 60 km from its source. It forms from the confluence between Pagliola (draining ASSM) and Cacarello Creeks, at the foot of the Monte Amiata (1738 m a.s.l., Tuscany). The catchment area (1320 km^2^ wide) is characterized by different rock types [34,35]: sedimentary rocks, belonging to several tectonic-stratigraphic units (Ligurids, Cervarola–Falterona–Trasimeno, Umbrian–Marchean Series), and magmatic rocks, attributable to two distinct volcanic districts (Monte Amiata and Monti Vulsini [36]). About 50% of the basin hosts agricultural or industrial activities; the other part is occupied by wooded or uncultivated areas [37,38]. The area has a Mediterranean (temperate) climate (hot and dry summer; cold and rainy winter). Stream sediments in Pagliola Creek reach Hg concentrations up to 8.4 mg kg^−1^ downstream from the mine of ASSM area [7].

Siele Creek, a right-side tributary of Paglia River, drains the south-eastern side of Monte Amiata. Its catchment covers an area of about 45 km^2^ (Figure 1B). It originates at an elevation of 710 m a.s.l., flows eastward for about 16 km, and then enters Paglia River near the regional border between Tuscany and Latium (Figure 1B). The upper sector of the Siele Creek hydrographic basin is largely occupied by Tuscan and Ligurian sedimentary formations (clay and limestone–marl flysch) [39]. The remaining portion of this waterway runs over the “Pliocene clays” [40], including marine, transitional, and continental sedimentary successions. Siele Creek has a torrential character and a mean slope of 8%, steeper in the first tract, while, downstream, the river is characterized by a larger waterbed, with a mean slope of 1%. The torrential regime of the upper part of the creek makes difficult to define the hydrological periods [41]. Powerful rainy events, floods, and flash floods are often causing associated sliding, such as mud- and debris-flows, in this area [42]. Siele Creek drains the Siele mining area (SMA), the oldest Hg mine in the MAMD. After closure in 1981, the area was subjected to the first reclamation in the district, completed in 2001. However, stream sediments in Siele Creek downstream of the mine still present highly anomalous Hg values up to 5400 mg kg^−1^ [23].

Stridolone Creek originates at an altitude of 860 m a.s.l. and drains the Cornacchino mining area (Figure 1B), closed in 1921 without any reclamation. It flows approximately in W–E direction for about 20 km to its confluence with Paglia River. In the first 5 km, it crosses the formations belonging to the Ligurian and Tuscan sequences (mainly marly limestones, argillites, and cherts [39]. In the upper section, the accentuated steepness and the torrential regime facilitate erosion. Landslides, mainly with sliding kinematics, with a moderate to high susceptibility to instability, are present particularly on the left bank. The central portion of the stream flows over Pliocene clays and is characterized by eluvium-colluvial layers. In the terminal part, volcanic and epiclastic rocks (mainly tuffs), associated with the Monte Amiata magmatic cycle [39], constitute the bedrock.

Agriculture is developed especially along the river stretch on Pliocene clays.

Rigo Creek is a left-side tributary of Paglia River (Figure 1), and it does not drain mine sites. Following previous work [43], it was selected to represent the regional geochemical background for Hg concentrations in water and sediments to compare with Hg at mine-affected sites. Accordingly, Hg in suspended matter of Paglia River is mainly in the form of sulphides (cinnabar or metacinnabar) [44] in accordance with that of stream sediments belonging to the Pagliola–Paglia system. Minor amounts of more soluble Hg species, such as HgCl_2_, HgO, and Hg^0^, were observed in waste calcines, soils, and stream sediments close to the mining area.

#### 2.1.2. Sampling Sites

The location of water sampling sites along the Paglia–Tiber River system is shown in Figure 1 and Table 1; in Figure 1, the location of bedload stream sediments sampling sites is also reported. At each site, water discharge was measured during sample collection.

Sites sampled in the September 2014 campaign included (Figure 1A):

- PC: at the Paglia River starting point at the confluence between Pagliola and Cacarello Creeks (same point sampled in 2020)
- PA: along Paglia River, near the town of Allerona, about 40 km from the source and 20 km upstream of the confluence with Tiber River AV: along Tiber River, downstream of the Alviano reservoir
- RM-PP: along Tiber River within the city limits of Rome
- RM-PG: along Tiber River downstream of the city of Rome

During early June and November 2020, campaigns’ water samples were collected at the following sites (Figure 1B,C):

Along Paglia River:

- PC: at Paglia River starting point (see above)
- TP2: downstream from confluence with Siele Creek

Along Siele Creek (Figure 1B,C):

- SIE_M: upstream of the SMA
- SIE_V: downstream of the SMA, at the exit of the mining site
- TSIE: downstream the SMA, where the steepness decreases, before the confluence with Paglia River

Along Stridolone Creek:

- TST: downstream of the Cornacchino mining area (Figure 1B)

Along Rigo Creek:

- RIGO: along Rigo Creek before the confluence with Paglia River. At this site, water was collected for total Hg (Hgt), and water discharge was not measured.

We must point out that development of field activities in 2020 was severely impacted by the COVID-19 pandemic. Notably, sampling was planned to represent comparatively low-flow conditions typical of summer in June 2020, whereas the November 2020 sampling campaign was expected to encounter comparatively high-flow conditions typical of autumn. As described below, those conditions were not met, and it was not possible to plan additional campaigns due to mobility limitations imposed by the pandemic.

Precipitation data recorded at the Piancastagnaio weather station in the period (eight days) before each sampling campaign are shown in Table 2. Autumn 2020 was characterized by a long period of draught. In June 2020, the eight days before sampling were characterized by four days of rain, with a peak of 65 mm day^−1^, and the wettest day of 2020 was recorded at the weather station. On the contrary, during the November campaign, rain was not recorded in the eight days before sampling. September 2014 was drier than June 2020.

### 2.2. Water Sampling Procedures

Water sampling for Hg was performed using “clean hands–dirty hands” procedures [46]. Water samples for total Hg (Hgt) were collected in acid precleaned (10% HCl) Teflon-lined glass bottles (100 mL) double-packed in plastic Ziploc bags. Samples for Hgt analysis were acidified (HCl 0.5% *v*/*v* Instrabaker) within 8 h of collection and then stored at 4 °C before analyses. An additional aliquot of water (2 L stored in pre-cleaned glass bottles) was collected to determinate Hg associated with particulate (Hgp) and dissolved species (Hgd). In June, the sampled water turned out too small for recovery of workable amounts of total suspended solids (TSS), and, consequently, Hgd and Hgp were not calculated.

### 2.3. Water Discharge Measurements

In 2014, water discharge (Q) at PC was measured by tracer dilution methods [47]. At AV, PA, and RM-PP, it was provided by the regional hydrographic services of Umbria and Latium, respectively. The discharge at RM-PG was assumed equal to RM-PP since there are no significant inflows between the two points (no monitoring station for discharge exists at this site).

During 2020 sampling campaigns, Q was measured using a magnetic flow meter (OTT MF PRO model) by the Regional Environmental Agency of Regione Toscana (ARPAT), following the UNI ISO 748 norm [48]. At site SIE_M, water flow was extremely low. In June, it was estimated by measuring the time required by water to fill a 5-L bucket. In November, the flow was practically nil, and it was not determined, nor were water samples taken.

### 2.4. Mercury Mass Load (M-Hg) Calculation

Mercury mass load (M-Hg; g day^−1^) is calculated as the product of Hg concentration (ng L^−1^) and the river discharge (L s^−1^) measured simultaneously. Mass loads were obtained for total (M-Hgt), dissolved (M-Hgd), and particulate Hg (M-Hgp). Since Hg concentrations, as well as stream water discharge, may change quite rapidly, mass loads are virtually an ephemeral parameter, especially during rainy periods or flash flood events. Nonetheless, we attempted extrapolation to daily loads based on the determined spot values.

### 2.5. Bedload Sediment Sampling

Stream bedload sediments were collected using bedload sediment traps. A Bunte Trap model was equipped with a filtering net of 100 µm opening size. A net with an opening size of about 1 cm was positioned at the entrance of the trap to prevent clogging by leaves or cobbles. Bedload traps were installed on the stream bed and anchored with metal stakes for variable periods (from few up to several weeks) to collect sediments transported along the riverbed and in suspension under different water fluxes. Bedload positioning was accomplished to (i) guarantee a continuous water flow through the trap; (ii) to have a position as close as possible to sampling points. Sampling was conducted between June 2018 and November 2020. The traps were positioned at seven sites (Figure 1B,C):

In Paglia River:

- AC1: at the Paglia River starting point
- TRAP_3: after the confluence with Minestrone Creek and before the confluence with Senna Creek

In Siele Creek:

- SIE_1: at the exit of Siele Creek from the mining area
- AC2 and SIE_3: at 400 and 550 m downstream the mining area, respectively
- AC3: about 1 km downstream of the TSIE sampling site

In Stridolone Creek:

- AC4: downstream the Cornacchino mining area

### 2.6. Chemical Analyses

Total mercury content (Hgt) in water samples collected in 2020 was analyzed at the MercuriLab in Trieste (Italy) by means of Cold Vapor Atomic Fluorescence Spectrometry (CV-AFS Mercury, Analytic Jena GmbH, Jena, Germany) [49].

Sample preparation for waters followed the EPA Method 1631 (2002) [50]. Water filtration was performed in laboratory by means of 0.45 µm sterilized pre-weighted cellulose membrane filters. After filtration, filters were dried at 60°C until a constant weight was achieved and then stored in a desiccator. The fraction of Hg associated with dissolved species (Hgd) was calculated as Hgt-Hgp. Mercury in the particulate fractions (see above for details about sampling) was analyzed by ARPAT following the EPA methods 3051A (2007) and EPA 6010D (2018) [51,52]. The same EPA 3051A 2007 and EPA 6010D 2018 methods were applied for the analysis of bedload sediments (see Fornasaro et al. [23] and Fornasaro et al. [25] for a full description of analytical methods).

## 3. Results

The water discharges (Q), Hg concentrations (Hgt, Hgd, and Hgp) in water, and calculated mass loads (M-Hg) obtained at the investigated sites are presented in Table 3. The bedload trap location and Hgt in bedload sediments are shown in Table 4.

### 3.1. Sampling Campaign 2014

**Figure 2:**
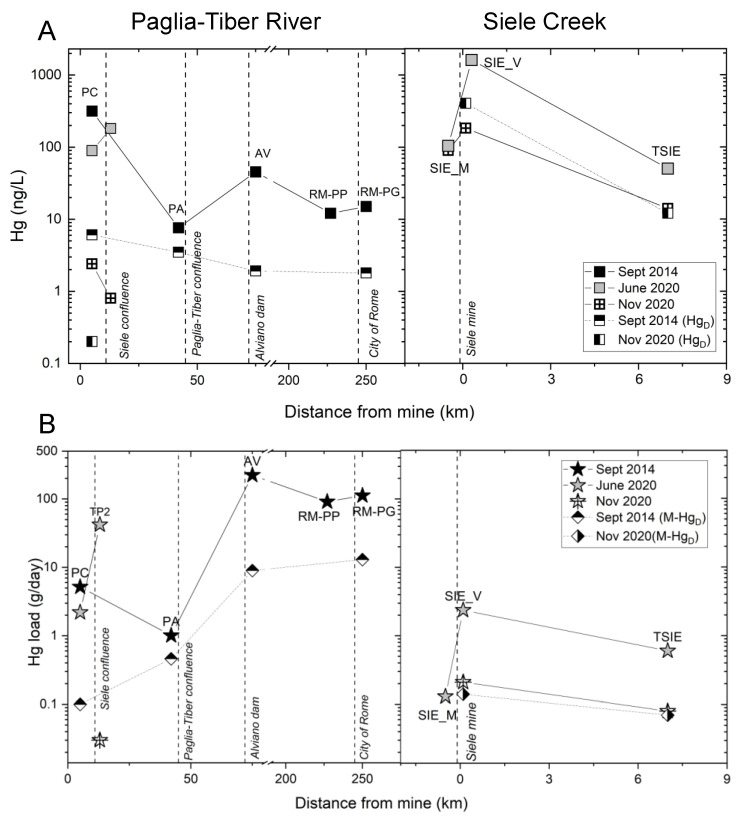
Variation in (**A**) Hgt concentrations (ng L^−1^) and (**B**) Hg mass loads (M-Hg) (g day^−1^) in the Paglia–Tiber River system and Siele Creek with distance from mine (km).

Water discharge along the Paglia–Tiber system (Table 3) showed the expected increasing trend from upstream (190 L s^−1^; PC) to downstream (88,700 L s^−1^; Rome). In the Paglia River basin, four days out of eight before sampling were considered “rainy”, with 51.8 mm of cumulate rain (Table 2). The total Hg content (Hgt) in water varied from a minimum of 8.0 ng L^−1^ (at PA) to a maximum of 316 ng L^−1^ (at PC). The dissolved Hg (Hgd) was considerably lower, ranging from 2.0 (at AV and RM-PG) to 6.0 ng L^−1^ (at PC) (Table 3, Figure 2A). The mercury mass load (M-Hgt) was 5 g day^−1^ at PC (Figure 2B) in the northern tract of Paglia River, where the watercourse enters an almost flat tract after its steep descent from ASSM. At PA (Allerona town), the M-Hgt reached its minimum value (~1 g day^−1^) after a gentle slope tract. Downstream from the Alviano reservoir (AV), the M-Hgt reached its maximum (220 g day^−1^), whereas, at Rome, the Hg mass load decreased to 90 g day^−1^ at RM-PP and 110 g day^−1^ at RM-PG (Table 3). The dissolved Hg mass loads (M-Hgd) showed an increasing trend from upstream to downstream. The lowest value is recorded at PC (0.1 g day^−1^) at the beginning of Paglia River and the maximum at RM-PG, close to the river mouth (13 g day^−1^).

### 3.2. Sampling Campaign 2020

In 2020, the water discharge measurements along Paglia River were higher in June than in November (Table 3). Correspondingly, the maximum Hgt (181 ng L^−1^) in stream water was recorded at the highest discharge (2700 L s^−1^) at TP2. In these conditions, the calculated M-Hgt reached its maximum, with 42 g day^−1^ of Hg load transported in water (Figure 2B). In the same season, the lowest M-Hgt value (2 mg day^−1^) was recorded at PC. In November, due to the drought period, Hgt remained low at all sites (<2.4 ng L^−1^), and it was mainly in the particulate form Hgp (2.2 ng L^−1^). Consequently, M-Hgt was almost negligible.

**Figure 3:**
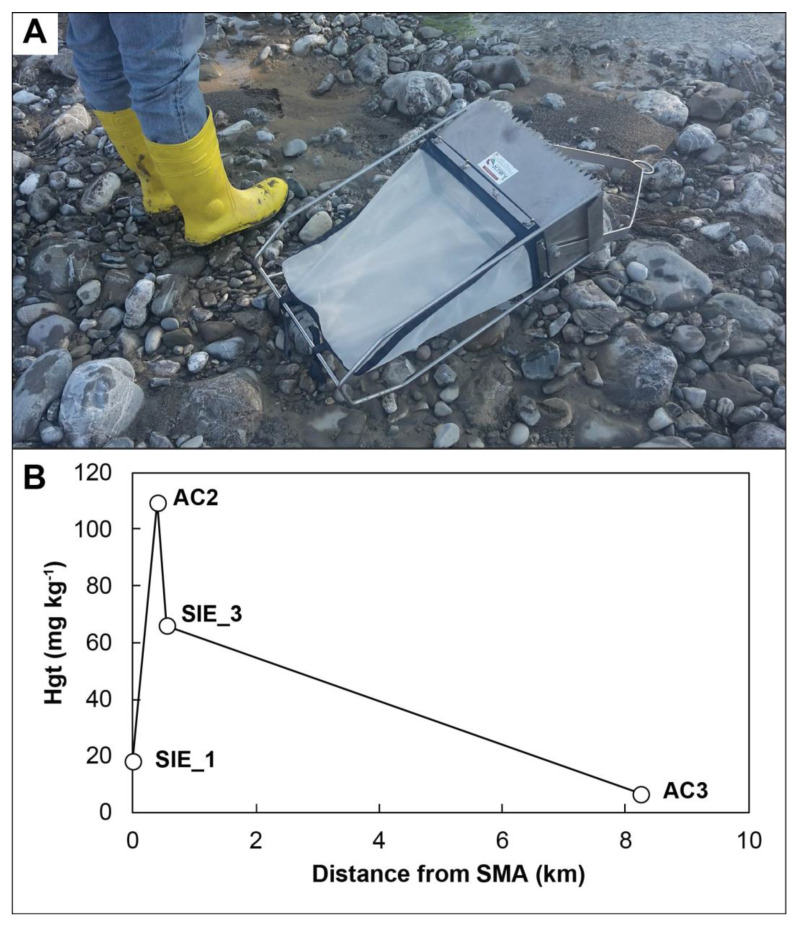
(**A**) Example of bedload trap (Bunte model). (**B**) Total Hg (mg kg^−1^) in bedload trap sediments vs. distance from the Siele mining area (SMA). Refer to Table 4 for the sampling periods.

Bedload sediments collected by the Bunte Trap (Figure 3A) were characterized by 2.0 mg kg^−1^ of Hg at AC1, and 23 mg kg^−1^ at TRAP_3 (Table 4).

Along Siele Creek, in June and November 2020, Q showed an increasing trend from upstream (SIE_M) to downstream (TSIE) (Table 2). At TSIE, in June, Q was higher (139 L s^−1^) than in November (63 L s^−1^). Similar to Paglia River, this variability is strongly related to the precipitation regime before the sampling campaigns (Table 2). In June, the Hgt in the water upstream from the mining area (SIE_M) varied from 103 ng L^−1^ to 1601 ng L^−1^ at the exit of the SMA (SIE_V), and it was 50 ng L^−1^ before the confluence with Paglia River (TSIE) (Figure 2A).

The mercury mass load (M-Hgt) upstream of the mine was 0.1 g day^−1^, and it increased at the outlet of the mining area (2 g day^−1^) and was lower at TSIE (0.6 g day^−1^) (Figure 2B).

In November, the Hgt measured in water was lower compared to June in connection with the lower water discharge (Table 3). The total Hg (Hgt) ranged from 14 (TSIE) to 603 (SIE_V) ng L^−1^ (Figure 2A), decreasing from upstream to downstream. The highest values are again observed at the exit of the Siele mining area (SIE_V), where both Hgt (603 ng L^−1^) and Hgd (401 ng L^−1^) reached their maximum (Figure 2A). Consequently, in this period, M-Hgt recorded a drastic reduction upstream and downstream (0.2 to 0.08 g day^−1^; Figure 2B).

Sediments collected in the bedload traps show highly variable Hg contents, ranging from 6.3 to a maximum of 110 mg kg^−1^ downstream from Siele mine (Table 4; Figure 3B).

Along Stridolone Creek, the water discharge was higher in November (32 L s^−1^) compared to June (11 L s^−1^); the same trend occurred for Hgt (November 38 ng L^−1^; June 18 ng L^−1^; Table 2). Correspondingly, in November, the M-Hgt was about 0.1 g day^−1^, five times higher than in June (Table 3). Mercury in the bedload trap along Stridolone Creek showed the highest values recorded in the MAMD area (290 mg kg^−1^; Table 4).

In Rigo Creek, the Hgt in the water collected in November was below the detection limit (1 ng L^−1^; Table 3), consistent with a previous measurement of 1.5 ng L^−1^ [43]. By contrast, in June, the water at the same location showed an unexpectedly high Hgt concentration (43 ng L^−1^), presumably because of the high turbidity following heavy rains in the preceding days (see Discussion).

## 4. Discussion

### 4.1. The Role of Water Discharge and Geohydrologic Setting on Hg Concentration and Mass Load

The concentration of Hgt found in the waterways of draining mines/smelting sites in the MAMD is generally high, confirming the data reported in previous works [43]. The results from two different years showed that the amount of Hg transported is highly variable, being strongly influenced by water discharge and by the local hydrologic conditions of the catchment area. During drought conditions, such as in November 2020, at PC, located immediately downstream of the MAMD district, and at TP2, at the Siele–Paglia confluence, Hgt is comparable to local background values (1.5 ng L^−1^), as found in Rigo Creek [43]. In June 2020, after a rainy event, water in Rigo Creek showed a higher concentration (43 ng L^−1^), suggesting that, during wet periods, Hg can also increase in waterways not draining mine sites.

This increase is most probably associated with the ubiquitous presence of Hg in the surrounding catchment, which can be transported via atmospheric deposition through dust or wind transport and then drained by water. A similar impact of local hydrologic conditions is observed at site PC, where, in September 2014, the Hgt was higher (316 ng L^−1^) at low water discharge than in June 2020 (89 ng L^−1^) during higher discharge (Table 3).

Along Siele Creek at a low water discharge, there is a corresponding low amount of Hgt. The strong influence of climate variability on Hg transport is also evidenced by the high Hg concentrations in bedload sediments along Siele Creek (110 mg kg^−1^) and Stridolone Creek (290 mg kg^−1^). These creeks display a torrential regime with discontinuous water discharge, strictly associated with rain events, implying that severe weather conditions control the physical transport of Hg-rich bedload sediments. Bedload sediments in Siele Creek and in Paglia River show decreasing Hg concentrations at further distance from the mine sites (Table 4). These data provide a qualitative overview of the transport of Hg in bedload sediments despite different sampling periods. It is to be considered that the bedload traps retain a relatively coarse sediment fraction (>100 µm); therefore, they do not include Hg associated with the <100 µm fraction and may, therefore, underestimate the total Hg transported in suspension with flowing waters.

Seasonal variations in the discharge regime show that the mass load of Hg delivered into Paglia River by its tributaries regarding draining mining and/or metallurgical districts responds to fluctuations in hydrological changes. For example, at TSIE (Siele Creek), M-Hgt was almost 10 times lower than along Paglia River (at PC) under similar water discharge regimes (139 L s^−1^ and 190 L s^−1^, respectively). On the other hand, in the initial part of Siele Creek (at SIE-V) M-Hgt was 2 g day^−1^ under low water discharge (17 L s^−1^, June 2020). This value is comparable to M-Hgt at PC in 2014 (5 g day^−1^) under higher discharge conditions (190 L s^−1^), showing how this small creek may become a substantial source of Hg despite its lower discharge compared to Paglia River. These results also suggest that the initial steep tract of Siele Creek may carry significant amounts of Hg. On the other hand, the final tract before the confluence with Paglia River (TSIE), characterized by a low steepness, behaves as a sink for Hg, particularly when water discharge is low. This behavior was indeed already observed and documented by Rimondi et al. [20] along Paglia. Actually, in the first steep tract (SIE-V), M-Hgt is high (2 g day^−1^, June 2020) but drops to 0.6 g day^−1^ a few kilometers downstream (at TSIE site), where the creek has an almost flat path. The same sites monitored in November 2020 provide a similar scenario (0.2 g day^−1^ at SIE-V vs. 0.08 g day^−1^ at TSIE). Consequently, under normal water discharge, a substantial part of Hg is deposited in the low steepness tract of the creek. It must be expected that heavy floods along the creek will mobilize Hg-bearing sediments, increasing the total M-Hgt and the overall Hg mass load from the MAMD through Paglia and Tiber Rivers and toward the Mediterranean Sea. Along Stridolone Creek (TST), the Hg mass loads are virtually negligible in both seasons under low discharge (0.02 and 0.1 g day^−1^, respectively). Notably, at TST, most of the Hg is in solution (Hgd) rather than associated with particulate, highlighting that, at a short distance from the mining/metallurgical centers, such as in Siele Creek (SIE_V), the concentration of dissolved Hg tends to be a significant fraction of Hgt.

Another important factor that controls Hg distribution along the Paglia–Tiber River system is the presence of dams. Gray et al. [51] and Rimondi et al. [7] documented that, along Tiber River, the Alviano reservoir behaves as a trap for Hg bound to particulate. However, we found that this trapping may be transitory. In September 2014, the Hgt in water collected immediately downstream from the dam (45 ng L^−1^; AV) was higher than Hgt upstream from the reservoir (8 ng L^−1^; PA). In those days, the Alviano reservoir was receiving important volumes of water from the upstream Corbara reservoir. The extremely high M-Hgt calculated downstream from Alviano (about 220 g day^−1^) is, thus, the result of a high water flux combined with a relatively high Hgt in the water.

In summary, the results (i) confirm that Hg contributions originating from the MAMD extend southward to Tiber River down to Rome and to its estuary, and (ii) suggest that the Tiber River section from Alviano to Rome may accumulate and transport Hg according to the river hydrologic conditions.

Overall, our data indicate that the Paglia–Tiber River waterway, including its tributaries, behaves as a transient—and, thus, unstable—trap for Hg. The effectiveness of the trap depends upon two main factors: (i) water discharge rate, and (ii) waterway steepness The higher these two factors, the higher the mass load of Hg is, highlighting that, at MAMD, hydrologic mobilization is a key driver of regional Hg cycling, also according to what is observed elsewhere (e.g., Refs. [53,54]). For example, more than 90% of the Hgt mass load in Artic rivers occurs during peak river discharge in spring and summer [53]. The combined effect of these two variables and the tendency of Hg to be transported physically, rather than chemically (with a few exceptions), triggers the deposition of Hg in insoluble phases along stretches with low steepness, where the energy provided by the flowing water is not high enough to suspend and transport Hg downstream. For example, along Paglia River, the Hgd in June 2014 and November 2020 represents less than 5% of the total Hgt (and up to 37%), and Hg is mainly transported in the particulate fraction rather than in solution, consistent with previous observations in Paglia River [20] and in other studies showing that, often, more than 90% of the total Hg content is usually transported in the form of suspended particulate matter [54,55,56].

Similarly, in Tiber River at RM-PG, in 2014, Hg was mainly associated with the particulate fraction, with higher Hgp compared to Hgd (13 ng L^−1^ compared to 2.0 ng L^−1^, representing 87% of the total Hg), and consistently lower Hgt compared to the upstream catchment. On the other hand, in Siele Creek, we observed that, at SIE_V and TSIE, dissolved mercury (Hgd) represents up to 86% of Hgt, although water discharge in this catchment controls Hg loads, as shown by comparing data at the same site in June and November 2020 (Table 3), when the water discharges were distinctly different. Dissolved Hg (Hgd) is high immediately after the tunnels that bypass the waste piles in the SMA, hinting at a local contribution of the mobile (water soluble) Hg fraction. The absence of data in the wet season makes it difficult to extrapolate this behavior to other periods of the year.

**Figure 4:**
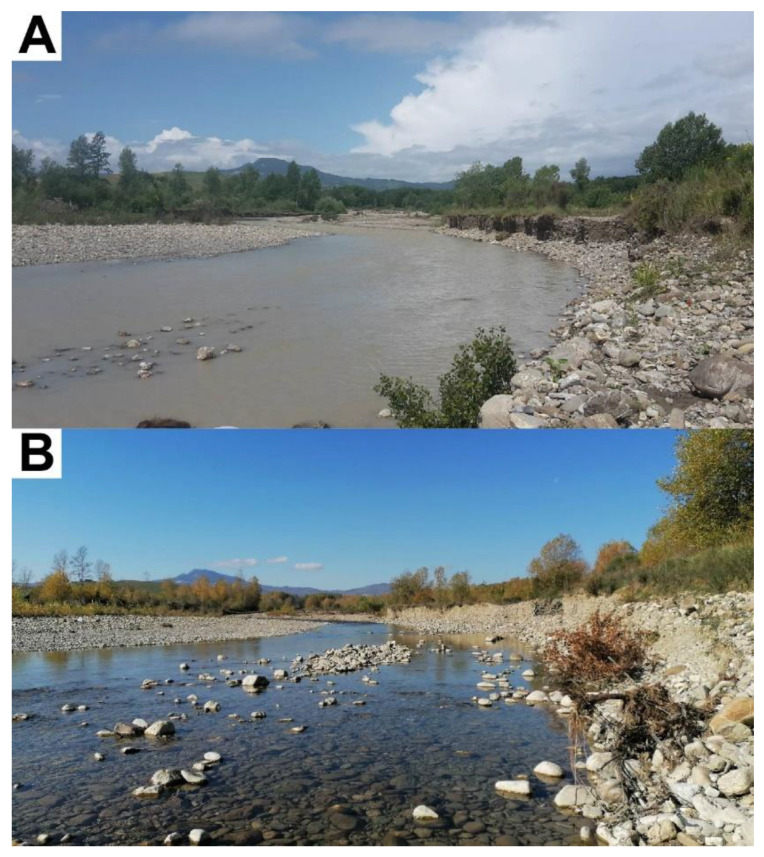
Water turbidity conditions in two different seasons at TP2 site. (**A**) In June 2020, after four intense rainy days; (**B**) in November 2020, after a period without significant precipitation.

In Paglia River, the prevailing association of Hg to suspended particulate can also be strictly linked to the fluvial depositional and geomorphologic history, together with the torrential regime of the upper catchment. The mobilization of old contaminated particles from the riparian and alluvial plain of Paglia River is enhanced after heavy rains or flood events [7,21,24], contributing to the recurrent distribution of Hg-contaminated legacy sediments in the floodplain and along the Paglia river course [24]. Not surprisingly, sediments collected in bedload traps are characterized by high concentrations of Hg (Table 4), confirming that Hg is actively transferred along all waterways of the MAMD, ultimately toward the sea. A qualitative example of different amounts of suspended particulate transported in Paglia River at the TP2 site in different weather conditions (June and November 2020) is shown in Figure 4. The highly turbid conditions during rainy periods highlight how these periodic events control the discharge of Hg associated with particulate along the river system, causing an intermittent input of Hg to Tiber River.

### 4.2. Contribution to Hg-Budget in the Mediterranean Sea

The results from the 2020 campaign provided an estimate of Hg discharge in the Paglia River catchment during two contrasting climatic conditions. In November, during a drought period, the Hg discharge in Paglia River was very low (0.03 g day^−1^), while, in June 2020, Paglia River discharged 40 g day^−1^ at the border between Tuscany and Latium (TP2), about 20 km before the confluence with Tiber River. This result is in good agreement with a value of 30 g day^−1^ calculated a few kilometers northward of PC during the wet season in 2011 [20].

Based on our data, the contributions to the mass load of Hg (M-Hgt) to Paglia River are at least 0.08–0.6 g day^−1^ from Siele Creek, and 0.02–0.1 g day^−1^ from Stridolone Creek. The total mercury load (M-Hgt) transported by Tiber River to the Mediterranean Sea at RM-PG and RM-PP in 2014 is 110 g day^−1^. Extrapolation to a yearly basis is problematic because of the extreme variability in calculated instant loads. The Hg load of 110 g day^−1^ estimated at Rome would correspond to about 40 kg year^−1^. This result is comparable to the mass loads of heavy metals (23 kg year^−1^) estimated by Montuori et al. [9] at the Tiber River estuary. Since there are no known major Hg sources along Tiber River, the elevated Hg mass load recorded in the final tract of the river suggests that the MAMD district is the main source of Hg reaching Rome, in agreement with previous work [20]. The slight increase found downstream from Rome (Figure 2) is presumably linked to urban sources in the estuary area.

Compared to other rivers draining Hg mines, the amount of Hg delivered by Tiber River to the Mediterranean Sea is similar to that transported by Guadalupe River (4–30 kg year^−1^) from the New Almaden Hg mines (California), the largest historic producers of Hg in North America [3]. On the other hand, it is considerably lower than the Hg discharged into the Adriatic Sea by Isonzo (Soča) River (about 1500 kg year^−1^), which receives waters from rivers draining the Idrija Hg mine in Slovenia [2]. Mass balance calculations suggest that up to ~39 × 10^3^ kg year^−1^ of cinnabar is transported by the Idrija River segment draining the Idrija mine before the confluence with Isonzo River to the Gulf of Trieste, under median flow conditions [55]. Remediation works at Abbadia San Salvatore and Siele, the main mine sites of the MAMD, are probably responsible for the lower Hg content discharged from Tiber River compared to the Idrija mine, where remediation never occurred.

The mercury load from Tiber River estimated here must take into account that the Paglia River system represents a permanent diffuse source of Hg-rich sediments, with at least 60 × 10^3^ kg of Hg stored in the overbank sediments and in the floodplain along the first tract of the Paglia River alluvial valley [22,24,25] and along its main tributaries [23]. Moreover, to the best of our knowledge, the amount of Hg stored in the Alviano reservoir and along the Tiber River stretch connecting this dam to the Mediterranean Sea is not known. As shown by our data, both the Alviano reservoir and Tiber River behave as a temporary sink for Hg, as well as sources of Hg (up to 223 g day^−1^ M-Hgt). The trapping effect of water reservoirs along rivers causes substantial fluctuations in contaminant releases downstream during dam operations or leakage (e.g., Refs. [57,58,59,60], representing an additional contribution to the intermittent release of Hg to the lower section of Tiber River. Moreover, the historical rainfall erosivity in the Tiber River basin drives hydrological events, with significant geomorphological effects [61]. Consequently, interannual hydrological variations, combined with the torrential regime of the upper catchment (Paglia River catchment), have a great impact on fluvial Hg transport, contributing to intermittent high Hg mass loads in Tiber River.

The variation in pollutants associated with particulate matter levels with hydrological conditions is highlighted in several studies (e.g., Refs. [11,24,62]), and the temporal variability in suspended particulate transported in contrasted watersheds (e.g., differing in discharge, steepness) was shown to be very high [63]. For example, the suspended particulate transport in a small mountainous river during a moderately dry year can be similar to that of a wet year in a large flat river, such as the Garonne [63]. Similarly, in Paglia River, as previously noted [20], and in its smaller tributaries, such as Siele and Stridolone Creeks, mass loads of Hg are highly variable between rainy and dry seasons. Unfortunately, here, we cannot compare the suspended particulate transported in different seasons and catchments because TSS values were only measured in November 2020. In the last few decades, changing climatic conditions triggered the Mediterranean area with shifts in climatic variability, with increasing heat waves, intense drought, and heavy rains [64,65,66]. In Central Italy (Tyrrhenian coast), an increase in drought events was recorded during the last 20 years, with the number of months under severe drought conditions increasing from 5% (1965–1999) to 24% (2000–2020) [65]. For example, in the Tiber River hydrographic district, the impact of an intense drought period from winter to early autumn 2017 also caused a decrease in water resources availability in the city of Rome [65]. Thus, the Hg loads estimated during these two different campaigns provide a quantitative picture of the Hg transport in Tiber River and its main tributaries during a drought and wet period, highlighting conditions expected to become very frequent. Indeed, further changes in climate conditions are predicted in the Mediterranean basin, with an increased frequency and severity of droughts and extreme precipitation, leading to variability in hydrological conditions (e.g., Refs. [66,67,68]). This recurrent weather variability may also alter the methylation capacity of freshwater bodies by creating environments favourable to methyl-Hg production (the extremely toxic and bioaccumulate form of Hg) (e.g., Ref. [53]), posing an additional risk to the fluvial environment. Previous works in the area found up to 8.7 ng/g of methyl-Hg in sediments downstream from the ASSM [43].

Consequently, updated data on the role of the MAMD district are essential to estimate the overall budget of Hg to the Mediterranean Sea through Tiber River, as well as to prevent potential risks for human health and the environment. Long-term observations covering interannual hydrological variations by monitoring the temporal variability in suspended particulate along the entire Paglia–Tiber River system will become necessary to accurately estimate fluctuations in Hg discharge in the river catchments and to the sea. Accurate and continuous measurements will also be useful to provide an estimate of the time-period discharge from the source of pollution, contributing to quantification of the future Hg budget into the Mediterranean Sea. Moreover, monitoring the trapping efficiency of the Alviano reservoir for Hg-contaminated sediments will help to determine the impact of dam water releases on Hg distribution in the lower segment of Tiber River and to quantify the storage and transport capacity of the river.

## 5. Conclusions

A detailed study of Hg mass loads in the upper Paglia River drainage network, combined with earlier data across the entire Paglia–Tiber River system, allowed an improved estimate of the annual Hg mass load delivered to the Mediterranean Sea (40 kg year^−1^). The main Hg source is the abandoned mining and smelting district of Monte Amiata (southern Tuscany), which is still delivering Hg to local waterways, in agreement with previous studies. Mercury transported through the drainage network, mainly associated with suspended particulate, tends to accumulate at the valley bottom, where rivers have gentler slopes, and is remobilized under high water discharge and transported downstream.

The results show that the spatial distribution of Hg transported by the Paglia–Tiber River system, as dissolved or particulate, is mainly controlled by the climatic conditions and catchment variability: the torrential regime in the upper catchment of Paglia River, the waterway steepness, and changing hydrologic conditions (water discharge rate). The mass load of total Hg in Paglia River varied from a minimum in November at PC (3 mg day^−1^) and a maximum in June at TP2 (42 g day^−1^), while, at the same site (PC), Hgt varied in different seasons (from 3 mg day^−1^ up to 5 g day^−1^). Along Tiber River, the Alviano reservoir showed a key role in the process of Hg storage/remobilization since most of the Hg-bearing particulate transported by Tiber River is accumulated in its bottom sediments. The storage of Hg in the reservoir is merely transient: floods and/or normal dam operations may mobilize high amounts of Hg (up to 223 g day^−1^), which are then transported by Tiber River towards the sea.

Estimated Hg loads provide a quantitative picture of Hg transport in the Paglia–Tiber River system during a dry and a wet period, highlighting conditions expected to become very frequent because of climate change. Intermittent Hg mass loads from the Paglia–Tiber River system are, therefore, expected to influence the Hg budget into the Mediterranean Sea in the future.

Considering these highly variable conditions, constant monitoring seems necessary to provide a continuous dataset. Permanent monitoring stations should be located along the Paglia and Tiber basin to outline the seasonal variability in the mass load of Hg also in relation to different hydrological situations.

Among the local-scale benefits, the full knowledge of the impacts of these hydrological events on Hg dynamics will help in the mitigation and management of Hg distribution from the MAMD through Tiber River. Such knowledge may also suggest a more efficient management of sediment through or around the Alviano reservoir to preserve the reservoir capacity and to minimize downstream impacts. Overall, the results will contribute to a more reliable estimate of the total Hg budget to the Mediterranean Sea, helping in finding global environmental management solutions.

Future work should, therefore, focus on quantifying Hg mass loads during high water discharge conditions in the upstream part of the Paglia–Tiber River system and at the estuary of Tiber River to provide a maximum estimate of Hg discharge into the Mediterranean Sea.

## Figures and Tables

**Table 1:** Location of the sampling sites.

Id	River/Creek	Latitude	Longitude	Campaign
PC	Pagliola	42.88291	11.72913	2014; 2020
TP2	Paglia	42.79536	11.80698	2020
PA	Paglia	42.77540	12.04620	2014
AV	Tiber	42.58313	12.25053	2014
RM-PP	Tiber	41.88342	12.47536	2014
RM-PG	Tiber	41.80527	12.34677	2014
SIE_M	Siele	42.78945	11.66759	2020
SIE_V	Siele	42.78944	11.66758	2020
TSIE	Siele	42.78700	11.74366	2020
TST	Stridolone	42.74460	11.76372	2020
RIGO	Rigo	42.82961	11.77992	2020

**Table 2:** Precipitation (mm day^−1^) at the Piancastagnaio weather station in the eight days before samplings. Data obtained from Ref. [45].

Period	9–16 September 2014	1–8 June 2020	29 October–5 November 2020
Cumulate	51.8	95.2	0.8
Daily Average	6.47	19.04	0.2
Raining days	4	4	0
Daily max	22	65	0.2

**Table 3:** Water discharge (Q, L s^−1^), total suspended solid (TSS, mg L^−1^), total Hg (Hgt, ng L ^−1^), dissolved Hg (Hgd, ng L^−1^), particulate Hg (Hgp, ng L^−1^), and Hg as total (M-Hgt), dissolved (M-Hgd), and particulate (M-Hgp) loads (g day^−1^). (*) assumed equal to RM-PP (see text); (**) measurements were conducted with a bucket.

	Q	TSS	Hgt	Hgd	Hgp	M-Hgt	M-Hgd	M-Hgp
	L s^−1^	mg L^−1^	ng L^−1^	ng L^−1^	ng L^−1^	g day^−1^	g day^−1^	g day^−1^
September 2014								
PC	190	-	316	6.0	310	5	0.1	5
PA	1530	-	8.0	3.0	4.0	1	0.5	0.6
AV	57,000	-	45	2.0	43	223	9	214
RM-PP	87,000	-	12	-	-	91	-	-
RM-PG	87,000 *	-	15	2.0	13	112	13	99
June 2020								
PC	285	-	89	-	-	2	-	-
TP2	2700	-	181	-	-	42	-	-
SIE_M	15 **	-	103	-	-	0.1	-	-
SIE_V	17	-	1601	-	-	2	-	-
TSIE	139	-	50	-	-	0.6	-	-
TST	11	-	18	-	-	0.02	-	-
RIGO	-	-	43	-	-	-	-	-
November 2020								
PC	13	0.5	2.4	0.2	2.2	0.003	0.0003	0.002
TP2	454	1.5	0.8	-	2.3	0.03	-	0.09
SIE_M	-	-	-	-	-	-	-	-
SIE_V	4	0.7	603	401	202	0.2	0.1	0.07
TSIE	63	0.2	14	12	2.3	0.08	0.07	0.01
TST	32	0.3	38	36	1.8	0.1	0.1	0.005
RIGO	-	-	<1	-	-	-	-	-

**Table 4:** Bedload trap location and total Hg (mg kg^−1^) concentrations in sediments collected in the bedload traps.

Id	Working Period	Hgt (mg kg^−1^)	Latitude	Longitude
Siele Creek				
SIE_1	June–October 2020	18	42.78961	11.66757
AC2	February 2019	110	42.78996	11.67208
SIE_3	June–July 2020	66	42.79017	11.67375
AC3	August 2019	6.3	42.78271	11.76357
Paglia River				
AC1	September 2018	2.0	42.88305	11.72879
TRAP_3	January–February 2020	23	42.82958	11.75200
Stridolone Creek				
AC4	February 2019	290	42.75318	11.67045

## Data Availability

Not applicable.

## References

[1] Amos H.M., Jacob D.J., Kocman D., Horowitz H.M., Zhang Y., Dutkiewicz S., Sunderland E.M. (2014). Global biogeochemical implications of mercury discharges from rivers and sediment burial. Environ. Sci. Technol..

[2] Širca A., Rajar R., Harris R.C., Horvat M. (1999). Mercury transport and fate in the Gulf of Trieste (Northern Adriatic)—A two-dimensional modelling approach. Environ. Model. Softw..

[3] Thomas M.A., Conaway C.H., Steding D.J., Marvin-DiPasquale M., Abu-Saba K.E., Flegal A.R. (2002). Mercury contamination from historic mining in water and sediment, Guadalupe River and San Francisco Bay, California. Geochem. Explor. Environ. Anal..

[4] Berzas Nevado J., Bermejo L.G., Martín-Doimeadios R.R. (2003). Distribution of mercury in the aquatic environment at Almadén, Spain. Environ. Pollut..

[5] Domagalski J., Majewski M.S., Alpers C.N., Eckley C.S., Eagles-Smith C.A., Schenk L., Wherry S. (2016). Comparison of mercury mass loading in streams to atmospheric deposition in watersheds of Western North America: Evidence for non-atmospheric mercury sources. Sci. Total Environ..

[6] Scanu S., Piazzolla D., Frattarelli F.M., Mancini E., Tiralongo F., Brundo M.V., Marcelli M. (2016). Mercury enrichment in sediments of the coastal area of northern Latium, Italy. Bull. Environ. Contam. Toxicol..

[7] Rimondi V., Costagliola P., Lattanzi P., Morelli G., Cara G., Cencetti C., Fagotti C., Torricelli S. (2019). A 200 km-long mercury contamination of the Paglia and Tiber floodplain: Monitoring results and implications for environmental management. Environ. Pollut..

[8] Cossa D., Coquery M., Saliot A. (2005). The Mediterranean Mercury Anomaly, a Geochemical or a BiologocalIssue. The Mediterranean Sea. Handbook of Environmental Chemistry.

[9] Montuori P., Aurino S., Garzonio F., Nardone A., Triassi M. (2016). Estimation of heavy metal loads from Tiber River to the Tyrrhenian Sea and environmental quality assessment. Environ. Sci. Pollut. Res..

[10] Rajar R., Četina M., Horvat M., Žagar D. (2007). Mass balance of mercury in the Mediterranean Sea. Mar. Chem..

[11] Poulier G., Launay M., Le Bescond C., Thollet F., Coquery M., Le Coz J. (2019). Combining flux monitoring and data reconstruction to establish annual budgets of suspended particulate matter, mercury and PCB in the Rhône River from Lake Geneva to the Mediterranean Sea. Sci. Total Environ..

[12] Rytuba J.J. (2003). Mercury from mineral deposits and potential environmental impact. Environ. Geol..

[13] Ferrara R., Maserti B.E., Andersson M., Edner H., Ragnarson P., Svanberg S. (1997). Mercury degassing rate from mineralized areas in the Mediterranean basin. Water Air Soil Pollut..

[14] Renzoni A., Zino F., Franchi E. (1998). Mercury levels along the food chain and risk for exposed populations. Environ. Res..

[15] Gray J.E., Theodorakos P.M., Bailey E.A., Turner R.R. (2000). Distribution, speciation, and transport of mercury in stream-sediment, stream-water, and fish collected near abandoned mercury mines in southwestern Alaska, USA. Sci. Total Environ..

[16] Faganeli J., Horvat M., Covelli S., Fajon V., Logar M., Lipej L., Cermelj B. (2003). Mercury and methylmercury in the Gulf of Trieste (northern Adriatic Sea). Sci. Total Environ..

[17] Covelli S., Piani R., Acquavita A., Predonzani S., Faganeli J. (2007). Transport and dispersion of particulate Hg associated with a river plume in coastal Northern Adriatic environments. Mar. Pollut. Bull..

[18] Canu D.M., Rosati G., Solidoro C., Heimbürger L.E., Acquavita A. (2015). A comprehensive assessment of the mercury budget in the Marano–Grado Lagoon (Adriatic Sea) using a combined observational modeling approach. Mar. Chem..

[19] Vaselli O., Higueras P., Nisi B., Esbrí J.M., Cabassi J., Martínez-Coronado A., Tassi F., Rappuoli D. (2013). Distribution of gaseous Hg in the Mercury mining district of Mt. Amiata (Central Italy): A geochemical survey prior the reclamation project. Environ. Res..

[20] Rimondi V., Costagliola P., Gray J.E., Lattanzi P., Nannucci M., Paolieri M., Salvadori A. (2014). Mass loads of dissolved and particulate mercury and other trace elements in the Mt. Amiata mining district, Southern Tuscany (Italy). Environ. Sci. Pollut. Res..

[21] Pattelli G., Rimondi V., Benvenuti M., Chiarantini L., Colica A., Costagliola P., Di Benedetto F., Lattanzi P., Paolieri M., Rinaldi M. (2014). Effects of the November 2012 flood event on the mobilization of Hg from the Monte Amiata Mining District to the sediments of the Paglia River Basin. Minerals.

[22] Colica A., Benvenuti M., Chiarantini L., Costagliola P., Lattanzi P., Rimondi V., Rinaldi M. (2019). From point source to diffuse source of contaminants: The example of mercury dispersion in the Paglia River (Central Italy). Catena.

[23] Fornasaro S., Morelli G., Rimondi V., Fagotti C., Friani R., Lattanzi P., Costagliola P. (2022). Mercury distribution around the Siele Hg mine (Mt. Amiata district, Italy) twenty years after reclamation: Spatial and temporal variability in soil, stream sediments, and air. J. Geochem. Explor..

[24] Fornasaro S., Morelli G., Rimondi V., Fagotti C., Friani R., Lattanzi P., Costagliola P. (2022). The extensive mercury contamination in soil and legacy sediments of the Paglia River basin (Tuscany, Italy): Interplay between Hg-mining waste discharge along rivers, 1960s economic boom, and ongoing climate change. J. Soils Sediments.

[25] Fornasaro S., Morelli G., Rimondi V., Fagotti C., Lattanzi P., Costagliola P. (2022). A GIS-based map of the Hg-impacted area in the Paglia River basin (Monte Amiata Mining District–Italy): An operational instrument for local authorities. J. Geochem. Explor..

[26] Lattanzi P., Benvenuti M., Chiarantini L., Colica A., Costagliola P. Mercury fluxes from the abandoned Monte Amiata mining district in the Paglia and Tiber River catchments, Central Italy: Preliminary estimates. Proceedings of the EGU General Assembly 2020.

[27] Directive W.F. (2000). Water Framework Directive. J. Ref. OJL.

[28] Camici S., Tarpanelli A., Brocca L., Melone F., Moramarco T. (2011). Design soil moisture estimation by comparing continuous and storm-based rainfall-runoff modeling. Water Resour. Res..

[29] Minissi S., Lombi E. (1997). Heavy metal content and mutagenic activity, evaluated by Vicia faba micronucleus test, of Tiber River sediments. Mutat. Res. Toxicol. Environ. Mutagen..

[30] Bettinetti R., Galassi S., Guilizzoni P., Quadroni S. (2011). Sediment analysis to support the recent glacial origin of DDT pollution in Lake Iseo (Northern Italy). Chemosphere.

[31] Patrolecco L., Capri S., Ademollo N. (2014). Occurrence of selected pharmaceuticals in the principal sewage treatment plants in Rome (Italy) and in the receiving surface waters. Environ. Sci. Pollut. Res..

[32] Girotti O., Mancini M. (2003). Plio-Pleistocene stratigraphy and relations between marine and non-marine successions in the Middle Valley of the Tiber River (Latium, Umbria). Il Quat..

[33] Cencetti C., De Rosa P., Fredduzzi A., Tacconi P., Cencetti C., Ruggiero D. (2020). L’approccio morfologico-sedimentario nello studio dei corsi d’acqua: Dinamica fluviale, processi di erosione, rischio da dinamica d’alveo e rischio idraulico. Atti del Convegno “Dal Fiume al Mare—Ripensare il Litorale Romano Secondo Natura” (Ostia, 12 Ottobre 2019). Collana “Culture, Territori, Linguaggi”.

[34] Boila P., Lavecchia G., Giaquinto S., Pialli G. (1982). Caratteri geologico-strutturali del bacino del Fiume Paglia (Umbria-Toscana). Progett. Final. Energetica CNR—Sottoprogetto Energ. Geoterm. (CNR). Relaz. Final..

[35] Damiani A.V., Mencarelli I. (1990). Controlli strutturali subiti dalla sedimentazione “etrusca” affiorante nella finestra tettonica del M. Peglia (Umbria di SW). Rend. Soc. Geol. It.

[36] Marra F., Costantini L., Di Buduo G., Florindo F., Jicha B., Monaco L., Palladino D., Sottili G. (2019). Combined glacio-eustatic forcing and volcano-tectonic uplift: Geomorphological and geochronological constraints on the Tiber River terraces in the eastern Vulsini Volcanic District (central Italy). Glob. Planet. Chang..

[37] Regione Toscana, 2019 Carta Dell’uso del Suolo, Scale 1:10.000. http://www502.regione.toscana.it/geoscopio/usocoperturasuolo.html.

[38] Regione Lazio, 2000 Carta Dell’uso del Suolo. Assessorato Urbanistica e Casa–Regione Lazio. Scala 1:25,000 Regione Lazio, Roma, Italy. https://dati.lazio.it/catalog/it/dataset/carta-tecnica-regionale-1979-1980-25k.

[39] Marroni M., Moratti G., Costantini A., Conticelli S., Benvenuti M.G., Pandolfi L., Laurenzi M.A. (2015). Geology of the Monte Amiata region, Southern Tuscany, Central Italy. Ital. J. Geosci..

[40] Ciccacci S., Galiano M., Roma M.A., Salvatore M.C. (2009). Morphodynamics and morphological changes of the last 50 years in a badland sample area of Southern Tuscany (Italy). Z. Geomorphol..

[41] Moretti G.P., Cianficconi F., Peroni E., Ronca M. (1988). Considerazioni sulle comunità macrobentoniche del sistema fluviale Paglia-Chiani. Boll. Mus. Sto. Nat. Lunigiana.

[42] Di Tria L., Grimaldi S., Napolitano F., Ubertini L. Rainfall forecasting using limited area models and stochastic models. Proceedings of the EGS Plinius Conference.

[43] Rimondi V., Gray J.E., Costagliola P., Vaselli O., Lattanzi P. (2012). Concentration, distribution, and translocation of mercury and methylmercury in mine-waste, sediment, soil, water, and fish collected near the Abbadia San Salvatore mercury mine, Monte Amiata district, Italy. Sci. Total Environ..

[44] Rimondi V., Bardelli F., Benvenuti M., Costagliola P., Gray J.E., Lattanzi P. (2014). Mercury speciation in the Mt. Amiata mining district (Italy): Interplay between urban activities and mercury contamination. Chem. Geol..

[45] Regione Toscana (2020). SIR, Settore Idrologico e Geologico Regionale. www.sir.toscana.it.

[46] US-EPA (1996). Method 1669: Technical Guidance-Sampling and Analysis of Ambient Waters for Trace Metals at EPA Water Quality Criteria Levels. https://www.epa.gov/sites/production/files/2015-10/documents/method_1669_1996.Pdf.

[47] Kimball B.A. (1997). Use of tracer injections and synoptic sampling to measure metal loading from acid mine drainage. U.S. Geological Survey Fact Sheet FS-245-96.

[48] (2008). Hydrometry—Measurement of liquid flow in open channels using current-meters or floats.

[49] Pavoni E., Petranich E., Signore S., Fontolan G., Covelli S. (2021). The Legacy of the Idrija Mine Twenty-Five Years after Closing: Is Mercury in the Water Column of the Gulf of Trieste Still an Environmental Issue?. Int. J. Environ. Res. Public Health.

[50] US-EPA (2002). Method 1631: Mercury in Water by Oxidation, Purge and Trap, and Cold Vapor Atomic Fluorescence Spectrometry. https://www.epa.gov/sites/production/files/2015-08/documents/method_1631e_2002.pdf.

[51] US-EPA (2007). Method 3051A (SW-846): Microwave Assisted Acid Digestion of Sediments, Sludges, and Oils, Revision 1. Washington, DC, USA. https://www.epa.gov/esam/us-epa-method-3051a-microwave-assisted-acid-digestion-sediments-sludges-and-oils.

[52] US-EPA (2018). Method 6010D (SW-846): Inductively Coupled Plasma-Optical Emission Spectrometry, SW-846. https://www.epa.gov/esam/epa-method-6010d-sw-846-inductively-coupled-plasma-atomic-emission-spectrometry.

[53] Gray J.E., Rimondi V., Costagliola P., Vaselli O., Lattanzi P. (2013). Long-distance transport of Hg, Sb, and As from a mined area, conversion of Hg to methyl-Hg, and uptake of Hg by fish on the Tiber River basin, west-central Italy. Environ. Geochem. Health.

[54] Zolkos S., Krabbenhoft D.P., Suslova A., Tank S.E., McClelland J.W., Spencer R.G., Holmes R.M. (2020). Mercury export from Arctic great rivers. Environ. Sci. Technol..

[55] Baptista-Salazar C., Biester H. (2019). The role of hydrological conditions for riverine Hg species transport in the Idrija mining area. Environ. Pollut..

[56] Salomons W., Förstner U. (1984). Sediments and the transport of metals. Metals in the Hydrocycle.

[57] Schäfer J., Blanc G., Audry S., Cossa D., Bossy C. (2006). Mercury in the Lot–Garonne River system (France): Sources, fluxes and anthropogenic component. Appl. Geochem..

[58] Wei L., Liu Y., Cai D., Li F., Luo D., Li C., Xu G., Xiao T., Wu Q., He H. (2022). River morphology redistributes potentially toxic elements in acid mine drainage-impacted river sediments: Evidence, causes, and implications. Catena.

[59] Rayne S., Friesen K. (2009). Contaminant trapping behind large Dams: A mini-Review. Nat. Preced..

[60] Castelle B., Bonneton P., Dupuis H., Sénéchal N. (2007). Double bar beach dynamics on the high-energy meso-macrotidal French Aquitanian Coast: A review. Mar. Geol..

[61] Diodato N., Ljungqvist F.C., Bellocchi G. (2021). A millennium-long climate history of erosive storms across the Tiber River Basin, Italy, from 725 to 2019 CE. Sci. Rep..

[62] Ollivier J., Töwe S., Bannert A., Hai B., Kastl E.M., Meyer A., Schloter M. (2011). Nitrogen turnover in soil and global change. FEMS Microbiol. Ecol..

[63] Coynel A., Schäfer J., Hurtrez J.E., Dumas J., Etcheber H., Blanc G. (2004). Sampling frequency and accuracy of SPM flux estimates in two contrasted drainage basins. Sci. Total Environ..

[64] Chiang F., Mazdiyasni O., AghaKouchak A. (2021). Evidence of anthropogenic impacts on global drought frequency, duration, and intensity. Nat. Commun..

[65] Romano E., Petrangeli A.B., Salerno F., Guyennon N. (2021). Do recent meteorological drought events in central Italy result from long-term trend or increasing variability?. Int. J. Climatol..

[66] Castellari S., Venturini S., Giordano F., Ballarin Denti A., Bigano A., Bindi M., Zavatarelli M. (2014). Elementi per una Strategia Nazionale di Adattamento ai Cambiamenti Climatici.

[67] Giorgi F., Lionello P. (2008). Climate change projections for the Mediterranean region. Glob. Planet. Chang..

[68] Vicente-Serrano S.M., Quiring S.M., Peña-Gallardo M., Yuan S., Domínguez-Castro F. (2020). A review of environmental droughts: Increased risk under global warming?. Earth-Sci. Rev..

